# A case report of complete remission of acute myeloid leukemia combined with *DNMT3A, FLT3-TKD*, and *IDH2* gene mutations and active pulmonary tuberculosis treated with homeharringtonine + venetoclax + azacytidine

**DOI:** 10.3389/fmed.2023.1180757

**Published:** 2023-06-13

**Authors:** Lin Ji, Wei Yang, Xiao-feng Xu, Ya-qing Xu

**Affiliations:** Department of Oncology and Hematology, Hangzhou Red Cross Hospital, Hangzhou, Zhejiang, China

**Keywords:** homeharringtonine, acute myeloid leukemia, active pulmonary tuberculosis, FLT3-TKD mutation, complete remission, DNMT3A mutation, IDH2 mutation

## Abstract

In March 2022, a 58-year-old man was admitted to the local hospital for nausea and vomiting. His blood routine indicated that he had leukocytosis and anemia. The patient was diagnosed with acute myeloid leukemia (AML)-M5b accompanied by *DNMT3A, FLT3-TKD*, and *IDH2* mutations, chest CT revealed pulmonary tuberculosis (TB). Acid-fast bacillus (AFB) was detected in sputum. The patient then received anti-TB treatment with isoniazid + rifampicin + pyrazinamide + ethambutol. On April 8, he was transferred to our hospital's Hematology Department after three consecutive negative sputum smears. He was administered the VA (Venetoclax + Azacytidine) regimen of anti-leukemia treatment and also received levofloxacin + isohydrazide + pyrazinamide + ethambutol anti-TB treatment. After one course of VA therapy, there was no remission in the bone marrow. Therefore, the patient received the HVA (Homeharringtonine + Venetoclax + Azacytidine) regimen of anti-leukemia treatment. On May 25, the bone marrow smear revealed that the original mononuclear cells were 1%. Moreover, bone marrow flow cytometry revealed the absence of any abnormal cells. mNGS showed DNMT3A (mutation rate 44.7%), but no mutations were detected in FLT3-TKD and IDH2. The patient then received the HVA regimen three consecutive times, resulting in complete remission. Repeated chest CT examinations revealed progressive regression of pulmonary TB foci, no AFB was detected in the sputum. This AML patient with DNMT3A, FLT3-TKD, and IDH2 mutations and active TB is difficult to treat. It is very necessary for him to administer prompt anti-leukemia treatment under the premise of active anti-TB treatment. The HVA regimen is effective for this patient.

## 1. Introduction

Acute myeloid leukemia (AML) is a highly heterogeneous group of hematopoietic malignancies characterized by abnormal clonal proliferation, impaired differentiation, and blocked apoptosis of myeloid hematopoietic stem cells. The most common mutations found in AML include FLT3, NPM1, IDH2, DNMT3A, NRAS, and compound mutations, respectively (1). Several clinical studies have demonstrated that the prognosis for AML patients with coexisting mutations is generally poor (2–5). The presence of concurrent DNMT3A, FLT3-TKD, and IDH2 mutations in this patient has not been previously reported in the literature, is rare, and is associated with a poor prognosis. China is a large country affected by tuberculosis (TB), and many leukemia patients also have concomitant TB (6–8). How to treat AML patients with active TB, which is more challenging in the clinic, is a topic worth exploring. Venetoclax + Azacytidine is currently indicated for AML patients who are not candidates for intensive chemotherapy and is a commonly used treatment for AML with FLT3 and IDH2 mutations (9–12). Homoharringtonine (HHT) is used as a major component of combination chemotherapy regimens for AML in China and is more effective against gene mutations like FLT3 (13–15). The following AML case also carried mutations in DNMT3A, FLT3-TKD, and IDH2 with active TB. This patient received prompt chemotherapy with the HVA (homeharringtonine + venetoclax + azacytidine) regimen under the premise of active anti-TB treatment. Moreover, the patient achieved excellent remission, creating an opportunity for bone marrow transplantation. Therefore, through the therapeutic research in this case, clinicians should be more aware of the diagnosis and treatment strategy for this type of patient and balance the relationship between anti-TB and anti-leukemia treatments.

## 2. Case presentation

A 58-year-old man was admitted to a local hospital in March 2022 due to nausea and vomiting. His blood test results showed that he had a white blood cell count (WBC) of 25.3 × 10^9^/L, hemoglobin (HB) 65 g/L, and platelet (PLT) 190 × 10^9^/L. At the time, the doctor considered the possibility of acute leukemia. He was then transferred to the Hematology Department of The First Affiliated Hospital, Zhejiang University School of Medicine. When the patient's bone marrow was examined, the findings revealed that there was clearly active mononuclear system hyperplasia, with 51% of original mononuclear cells and immature mononuclear cells. Bone marrow flow cytometry indicated that the original myeloid cell population accounted for 20.46% of non-erythroid cells, expressing CD117, CD34, CD33, CD13, HLA-DR, CD38, CD123, and CD56. The chromosome was a normal karyotype. *BCR/ABL, AML1/ETO*, and *PML/RaR*α*fusion* genes were all negative. DNMT3A (mutation rate 48.02%), FLT3-TKD (mutation rate 36.3%), and IDH2 (mutation rate 45.95%) were found by next-generation sequencing (NGS). The patient was diagnosed as having acute myeloid leukemia (AML)-M5b with DNMT3A, FLT3-TKD, and IDH2 mutations. Following the admission, the patient's general condition was assessed and symptomatic supportive care was intensified. But chest computed tomography (CT) revealed two pulmonary tuberculosis (TB). In the sputum, acid-fast bacillus (AFB) 3+ was found. The patient received 2 months of TB pleurisy treatment 30 years ago and then stopped taking tuberculous drugs.

**Figure 1:**
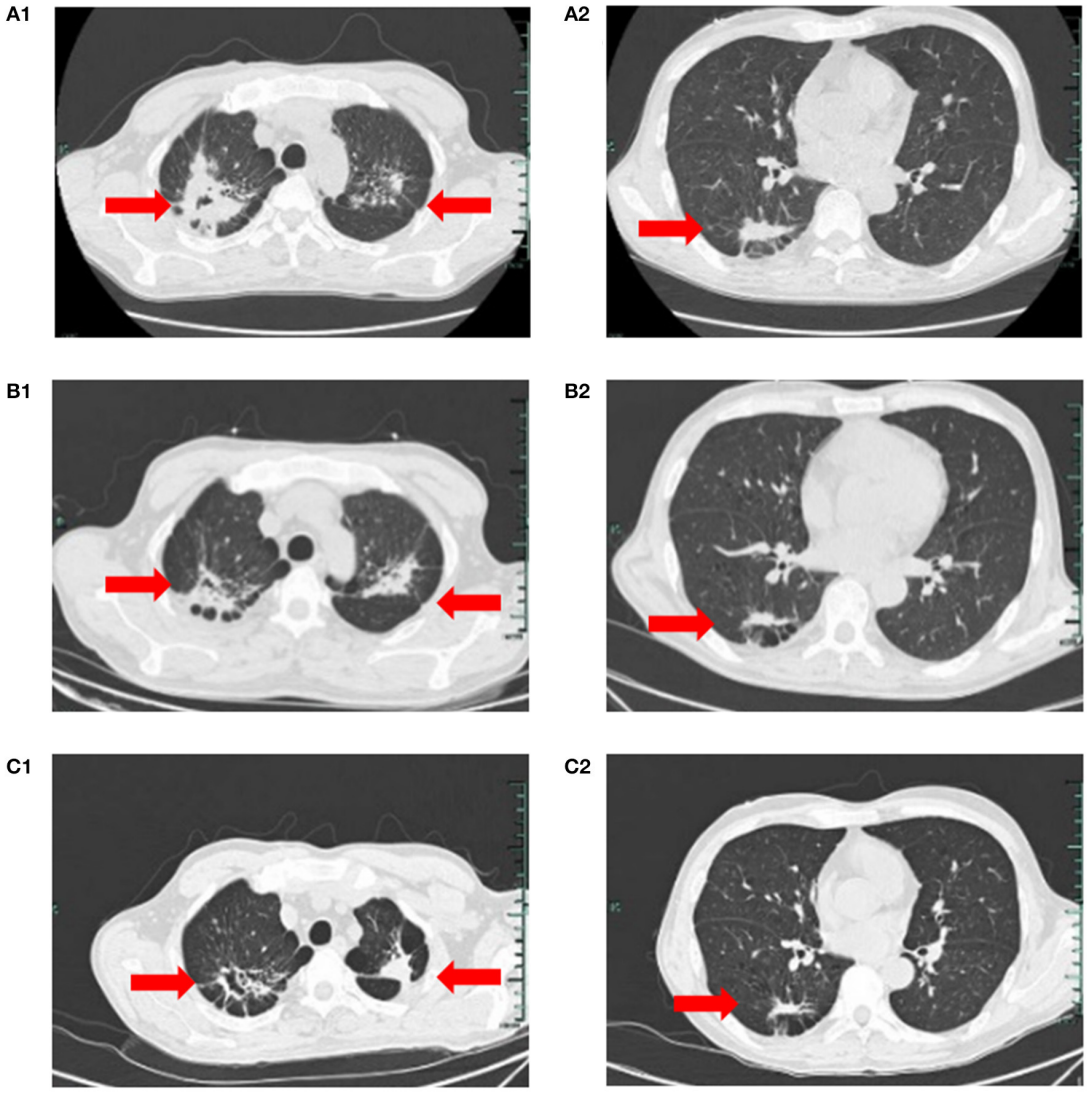
**(A1, A2)** On March 23, chest CT suggested speckled and patchy high-density shadows can be seen in both lungs, with unclear boundary, and cavity shadow can be seen in the upper right focus; Right pleural cord adhesion. **(B1, B2, C1, C2)** on July 5, 2022, and on August 3, 2022, respectively reviews of chest CT suggested the lung TB foci progressive reduction.

On March 23, the patient was transferred to the TB Department of our hospital. Blood counts were WBC 29.9 × 10^9^/L, neutrophils 17.9 × 10^9^/L, HB 60 g/L, PLT 175 × 10^9^/L, with manual classification showing abnormal cells in 15%. Two pulmonary TB lesions were visible on a chest CT, and the right upper lung cavity had formed (Figures 1A1, A2). The patient immediately received HRZE (Isohydrazide 0.3 g QD + Rifampicin 0.45 g QD + Pyrazinamide 1.5 g QD + Ethambutol 0.75 g QD) regimen anti-TB therapy. The patient's WBC did, nevertheless, reach a maximum of 99 × 10^9^/L, and neutrophils reached 59.8 × 10^9^/L. He was treated with hydroxycarbamide for the purpose of reducing his WBC count. When sputum smears were negative for 3 consecutive times, blood counts were WBC 11.9 × 10^9^/L, neutrophils 7.8 × 10^9^/L, HB 52 g/L, and PLT 74 × 10^9^/L.

On April 8, he was transferred to the Hematology Department of our hospital and started receiving VA (Venetoclax 100 mgd1, 200 mg d2–7+Azacytidine 100 mgd1–7) regimen anti-leukemia therapy immediately. Due to venetoclax and rifampicin conflict, the regimen of anti- TB regimen was adjusted to Levofloxacin 0.5 g QD + Isohydrazide 0.45 g QD + Pyrazinamide 1.5 g QD + Ethambutol 0.75 g QD. Following one course of the VA regimen, the bone marrow routine showed 4% primitive mononuclear cells and 35% naive mononuclear cells. A total 28% of juvenile mononuclear cells were identified by bone marrow flow cytometry. The chromosome was a normal karyotype. NGS highlighted DNMT3A (mutation rate 45.6%), FLT3-TKD (mutation rate 26.7%) and IDH2 (mutation rate 32.9%). On May 6, the patient was given HVA (Homoharringtonine 2 mg d1–7 + Venetoclax 100 mg d1, 200 mg d2–7 + Azacytidine 100 mg d1–7) regimen anti-leukemia therapy. On May 25, the blood counts routine showed WBC 5.2 × 10^9^/L, neutrophils 4.7 × 10^9^/L, HB 64 g/L, and PLT 336 × 10^9^/L. The original mononuclear cells in the bone marrow routine were only 1%. Flow cytometry of bone marrow showed no blasts with obvious immunophenotypic abnormalities. NGS test exhibited DNMT3A (mutation rate 44.7%), and no mutation was detected in IDH2 and FLT3-TKD. After that, the patient underwent three cycles of the HVA regimen anti-leukemia therapy consecutively on June 1, July 8, and August 9, and no abnormalities were found in multiple bone marrow routines and flow cytometry. On August 23, NGS showed DNMT3A (mutation rate 46.5%), with no mutation detected in IDH2 and FLT3-TKD. Moreover, on July 5, and on August 3, respectively reviews of chest CT suggested the lung TB foci progressive reduction (Figures 1B1, B2, C1, C2). No AFB was repeatedly detected in the sputum. He was treated with intrathecal chemotherapy three times (cytarabine 50 mg + methotrexate 15 mg + dexamethasone 5 mg) for the purpose of preventing the growth of central leukemia. On September 15, the blood routine showed WBC 4.1 × 10^9^/L, neutrophils 2.63 × 10^9^/L, HB 121 g/L, and PLT 242 × 10^9^/L. The patient has now had a bone marrow transplant. The adverse reactions of the patient during the use of HVA regimen are shown in Table 1.

**Table 1:** Adverse reactions during the use of HVA regimen.

**HVA regimen cycles**	**Adverse reactions in the blood system**				**Adverse reactions in the liver and kidney system**		**T_max_ (°C)**	**Is there a tumor lysis syndrome present**
	**PLT**_min_ **(10**^9^**/L)**	**WBC**_min_ **(10**^9^**/L)**	**N**_min_ **(10**^9^**/L)**	**HB**_min_ **(g/L)**	**ALT**_max_ **(U/L)**	**Cr**_max_ **(**μ**mol/L)**		
First	22	0.3	0.03	51	22	62.3	38.3	NO
Second	75	0.9	0.6	48	19	56.4	38.4	NO
Third	100	2	1.14	58	21	68.7	37.9	NO
Fourth	13	0.4	0.1	77	44	73.7	37.6	NO

Platelet (PLT) reference value: 100–300 × 10^9^/L; white blood cells (WBC) reference value: 3.5–9.5 × 10^9^/L; neutrophil (N) count reference value: 1.80–6.40 × 10^9^/L; hemoglobin (HB) reference value: 120–170 g/L; alanine transaminase (ALT) reference value: 9–50 U/L; creatinine (Cr) reference value: 41.0–111.0 μmol/L; min, minimum; max, maximum.

## 3. Discussion

AML is a highly heterogeneous group of hematopoietic malignancies characterized by abnormal clonal proliferation, impaired differentiation, as well as blocked apoptosis of myeloid hematopoietic stem cells. FLT3, NPM1, IDH2, DMNT3A, NRAS, and complex mutations were the most prevalent gene mutations in AML patients (1). Multiple clinical studies have claimed that NPM1, DNMT3A, and FLT3-ITD co-occur more frequently in AML patients and that the overall prognosis of AML patients with these three mutations co-exists is poor (2, 3). A worse prognosis is associated with patients who have both IDH2 and DNMT3A mutations (4, 5).

Although small molecules targeted agents have been developed recently for DNMT3A mutation AML, further research is still needed to determine the clinical outcome (16, 17). Targeted therapy is mainly used for AML patients with FLT3 mutation and IDH2 mutation. Type I FLT3 inhibitors, which can bind to the active and inactive conformation of FLT3, are multichines inhibitors that is not only effective against FLT3-ITD, but also against FLT3-TKD mutations, such as the first-generation inhibitors midostaurin, sunitinib, and the second-generation inhibitors giletitinib, crenolanib. But these drugs are expensive, and in China, most patients cannot afford the huge drug costs incurred during the long-term treatment of targeted drugs. Type II inhibitors can only bind to the inactive conformation, The first generation of sorafenib, ponatinib and the second generation of quizartinib are type II inhibitors, which being highly selective and strongly inhibitory against FLT3-ITD but weaker against FLT3-TKD (18–20). Enasidenib can stimulate megakaryocyte and erythroid progenitor differentiation and reverse the DNA methylation effect of IDH2 mutations (21). However, the drug is not on the market in China.

Multiple studies have claimed (9, 10) that Venetoclax and azacitidine combination modality can synergistically induce apoptosis, synergistically activate mitochondrial apoptosis in AML cells, and lower MCL-1 levels, which enhances patient tolerance to treatment. An inhibitor of enasidenib induces BCL-2 dependence, rendering them more sensitive to venetoclax (11). Venetoclax has been demonstrated to increase antitumor activity in combination with quizartinib, particularly in patients with FLT3 ITD mutated AML; In combination with gilteritinib, the survival of patients with FLT3 or DNMT3A mutations was improved (12). Hence, BCL-2 inhibitors in combination with demethylating agents or targeted agents make up the majority of the treatment regimens for patients with DNMT3A, FLT3, and IDH2 mutations.

The patient with concurrent mutations in three genes (*DNMT3A, FLT3-TKD*, and *IDH2*) with active TB is at extremely high risk for AML in which conventional chemotherapy regimens are largely myelosuppressive, and is detrimental to active TB control. Clinicians must therefore find an anti-leukemic therapy regimen with high efficiency and low toxicity. With low doses of BCL-2 inhibitors, 58.3% of AML patients in a large real-world study from India (22) were able to achieve CR or CRi, and the study also made the suggestion that shortening the course of BCL-2 inhibitors may achieve similar efficacy as the 28 days recommended regimen and a considerably shorter duration of granulocytic deficiency. In China, it has also been shown clinically that the use of a low-dose, short-course VA regimen allows patients to achieve Cr/CRi, but the findings have not been formally reported. In addition, Ramon-Luing's study shows that Mycobacterium Tuberculosis increases the expression of anti-apoptotic BCL-2 (13). So, the patient received an initial anti-leukemic therapy regimen as a low-dose short course of Venetoclax combined with Azacytidine. However, the patient still had 28% promonocyte on re-examination of bone marrow flow after completing one course of VA regimen, and DNMT3A, FLT3-TKD, and IDH2 mutations were still detected. It is clear that the patient cannot benefit from this regimen. For further treatment, protocol considerations require the addition of targeted agents or chemotherapeutic agents. But the huge drug costs incurred during long-term treatment with targeted drugs are beyond the reach of the majority of patients in developing countries. A chemotherapeutic drug that can be multitargeted, highly efficacious, and cost-effective is thus required.

Homoharringtonine (HHT), is used as a key component of combination chemotherapy regimens for AML and has been extensively utilized for over 30 years, in China. Experimental studies have found that HHT enhances the proapoptotic effects of venetoclax by lowering the expression of myeloid cell leukemia-1 (MCL-1) (14), and clinical studies have revealed that combination therapy of HHT with venetoclax and AZA has a better therapeutic response in R/R-AML (15). FLT3 is a crucial direct target of the HHT-TET1/5hmC axis in patients with FLT3 mutated AML, and AML cells with FLT3 mutation, and patients are very sensitive to HHT treatment (6). However, how effective HHT is specifically for FLT3-ITD and FLT3-TKD has yet to be clearly reported in the literature. Therefore, in this patient, we attempted to add HHT to the original regimen, which constituted the HVA regimen. After one course of treatment with the HVA regimen, the patient's bone marrow achieved better remission, and only a DNMT3A mutation was detected, with the goal of providing the patient with a bone marrow transplant. So, it was thought that HHT might have a synergistic anti-leukemia effect after combining the VA regimen, and it was speculated that HHT might also have better efficacy for FLT3-TKD mutation.

According to WHO data, in 2020, there are 842,000 new TB patients in China, representing an incidence of 8.5% in the world (7). The average prevalence of leukemia combined with TB in China is 3.9%, which is 8.5 times higher in comparison to the prevalence of active TB in the normal population (8). Studies have argued that patients who develop malignancies after a history of TB have a higher risk of TB reactivation (23). The patient has a higher risk of developing active pulmonary TB after having AML because he had tuberculous pleurisy 30 years ago.

The patient was treated with active anti-TB therapy upon admission; the timing of the initiation of anti-leukemia therapy is not determined. It has been reported that (24) 13 patients with acute leukemia combined with TB at the first diagnosis all delayed the first induction chemotherapy for 2 months, and eventually TB was controlled and leukemia was in remission; 2 patients with acute leukemia in combination with TB at the first diagnosis and delayed the first induction chemotherapy for 2 months failed to achieve remission and died. However, this case is particularly high-risk for leukemia and delayed anti-leukemia therapy could also impact TB control. After 2 weeks of anti-TB treatment, the patient's sputum smear results were consistently negative, thus he was treated with caution against leukemia. Meanwhile Venetoclax, in combination with rifampicin, may cause accelerated metabolism and lower efficacy (25), so the anti-TB regimen was adjusted prior to the patient starting anti-leukemia therapy. Sputum smears were negative on multiple subsequent reexaminations, and multiple chest CTs indicated gradual resorption of TB cavitation in both lungs, demonstrating the safety and efficacy of this anti-TB regimen. Therefore, for high-risk AML patients with active pulmonary TB, patients may benefit from timely anti-leukemic treatment under the premise of an active anti-TB regimen. You can treat TB first, then leukemia, for chronic or indolent leukemia. Such cases, however, were not numerous, and more cases need to be accumulated for further research.

In conclusion, the clinical treatment of AML patients with *DNMT3A, FLT3-TKD*, and *IDH2* gene mutations with active pulmonary TB is challenging, and timely anti-leukemia therapy is needed under the premise of active anti-TB therapy. The HHT + Venetoclax + azarctidine has a good therapeutic effect on the AML patient with *DNMT3A, FLT3-TKD*, and *IDH2* gene mutations, creating conditions for bone marrow transplantation.

## Data availability statement

The original contributions presented in the study are included in the article/supplementary material, further inquiries can be directed to the corresponding authors.

## Ethics statement

The studies involving human participants were reviewed and approved by Ethics Committee of Hangzhou Red Cross Hospital. The patients/participants provided their written informed consent to participate in this study. Written informed consent was obtained from the participant/patient(s) for the publication of this case report.

## Author contributions

X-fX and Y-qX were mainly responsible for the diagnosis and treatment of the patient. LJ and WY were mainly responsible for writing the case report. We have obtained patient consent to treatment. All authors contributed to the article and approved the submitted version.
